# Quantitative indices of autophagy activity from minimal models

**DOI:** 10.1186/1742-4682-11-31

**Published:** 2014-07-06

**Authors:** Kyungreem Han, Jinwoong Kim, MooYoung Choi

**Affiliations:** 1 Department of Physics and Astronomy and Center for Theoretical Physics, Seoul National University, Seoul 151-747, Korea; 2 College of Pharmacy and Research Institute of Pharmaceutical Sciences, Seoul National University, Seoul 151-742, Korea

**Keywords:** Autophagy, Quantitative indices, Minimal autophagy model, Computer simulations

## Abstract

**Background:**

A number of cellular- and molecular-level studies of autophagy assessment have been carried out with the help of various biochemical and morphological indices. Still there exists ambiguity for the assessment of the autophagy status and of the causal relationship between autophagy and related cellular changes. To circumvent such difficulties, we probe new quantitative indices of autophagy which are important for defining autophagy activation and further assessing its roles associated with different physiopathological states.

**Methods:**

Our approach is based on the minimal autophagy model that allows us to understand underlying dynamics of autophagy from biological experiments. Specifically, based on the model, we reconstruct the experimental context-specific autophagy profiles from the target autophagy system, and two quantitative indices are defined from the model-driven profiles. The indices are then applied to the simulation-based analysis, for the specific and quantitative interpretation of the system.

**Results:**

Two quantitative indices measuring autophagy activities in the induction of sequestration fluxes and in the selective degradation are proposed, based on the model-driven autophagy profiles such as the time evolution of autophagy fluxes, levels of autophagosomes/autolysosomes, and corresponding cellular changes. Further, with the help of the indices, those biological experiments of the target autophagy system have been successfully analyzed, implying that the indices are useful not only for defining autophagy activation but also for assessing its role in a specific and quantitative manner.

**Conclusions:**

Such quantitative autophagy indices in conjunction with the computer-aided analysis should provide new opportunities to characterize the causal relationship between autophagy activity and the corresponding cellular change, based on the system-level understanding of the autophagic process at good time resolution, complementing the current in vivo and in vitro assays.

## Background

Macroautophagy (hereafter referred to as autophagy) is a key homeostatic mechanism for the turnover of such intracellular components as proteins/organelles [1], and is further related to various human diseases such as cancer, metabolic disorders, and neurodegenerative diseases [2-7]. This has brought forth a number of physiological and molecular-level studies of autophagy in the last decades.

In the process of autophagy, abnormal and/or resident proteins/organelles degrade into representative metabolic/energy precursor molecules, amino acids and ATP, which can be used as new building blocks and energy sources, respectively. Specifically, the autophagic process begins with the formation of initial double-membrane structures called autophagosomes containing sequestered proteins/organelles. Then the autophagosomes fuse with endosomes/lysosomes to form autolysosomes. Finally, the contents engulfed in the autolysosomes are hydrolyzed via intralysosomal hydrolysis.

**Figure 1:**
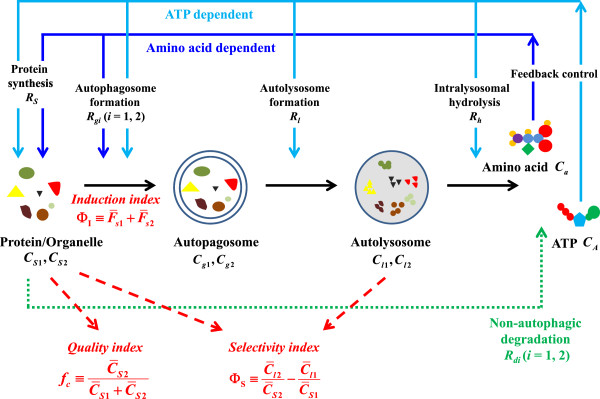
**Schematic representation of the model system and quantitative indices.** The model assumes a three-compartment description of the autophagic process: protein/organelle, autophagosome, and autolysosome compartments. Solid and dotted arrows denote the autophagic feedback loops among the compartments and non-autophagic degradation, respectively. The autophagy-related rates, including protein/organelle synthesis *R*_*S*_(*C*_*A*_, *C*_*a*_), autophagosome formation *R*_*g*1_(*C*_*A*_, *C*_*a*_) and *R*_*g*2_(*C*_*A*_, *C*_*a*_), autolysosome formation *R*_*l*_(*C*_*A*_), intralysosomal hydrolysis *R*_*h*_(*C*_*A*_), and non-autophagic degradation *R*_*d*1_ and *R*_*d*2_ are described in Appendix A. Dashed arrows indicate the quantitative indices *f*_*c*_, Φ_S_, and Φ_I_ of protein/organelle quality, autophagy selectivity, and autophagy induction, respectively.

The operation mechanism of the process is extremely complex. The three consecutive steps, i.e., autophagosome formation, autolysosome formation, and intralysosomal hydrolysis, are operated independently, exhibiting qualitatively/quantitatively different responses to different intra- or extra-cellular perturbations. Hence, assessment of the autophagy activity via specific markers such as autophagosomes and autolysosomes often leads to under-/over-estimation of the autophagy activity. For example, as revealed in experiment [8], if the rate of autolysosome formation exceeds that of autophagosome formation, the steady-state concentration of autophagosomes could be detected less, leading to an underestimation of the autophagy status. Moreover, the autophagic process is regulated by the complex positive–negative feedback mechanism (see Figure 1): The output of the process, i.e., recycled amino acids and ATP, acts as the input of the system, which independently manipulates each of the steps in a concentration-dependent manner [9-14]. Such feedback loops may be beneficial for the delicate balance of the cellular homeostasis. In the perspective of causality analysis/ assessment, however, it could lead to difficulties in interpreting the cause-and-effect relationship between the autophagy activity and the concomitant cellular change.

Even if those uncertainties in the assessment of the status and causality of autophagy are removed, there still remains ambiguity as to the roles of autophagy in the human disease since autophagy exhibits dual effects on the development and progression of various human diseases in a context- and activation-degree-dependent manner. Especially, autophagy plays a dual role in the tumor cell viability [15]: In some cases autophagy prevents or suppresses tumor progression whereas in other cases autophagy can also accelerate tumorigenesis or promote the survival of tumor cells. Under energy-deficient conditions, autophagy is usually activated for the rapid supply of essential energy/metabolites to promote cell survival. In contrast, it is not rare that induced autophagy contributes to apoptosis/necrosis under various cellular perturbations other than the energy deficiency. Accordingly, it is intriguing to interpret the newly elucidated mechanism of autophagy: whether the induction of autophagy contributes to the prevention of the disease and how it works. In particular, evolutionally new molecular-level studies sometimes contradict existing studies.

Based on the recent molecular- and physiological-level studies of the mechanism and the role of autophagy in the human disease, on the other hand, there is research being carried out on the development of the treatment methods or drugs that can potentially regulate or control autophagy [16-19]. In view of these, it is very desirable to develop specific and quantitative indices based on adequate mathematical model which could provide a set of reliable criteria for defining the autophagy status and further the kinetics of the process. To date, various indices based on biochemical [20-25] and morphological methods [26-30] for the detection of the autophagic sequestration of proteins/organelles and for the measurement of the turnover of autophagic compartments and/or autophagy-related markers have been developed for the assessment of autophagy. However, those indices are not quite satisfactory for the specific and quantitative assessment of the autophagy pathway as well as the system-level interpretation of the process, especially in mammalian cells.

**Figure 2:**
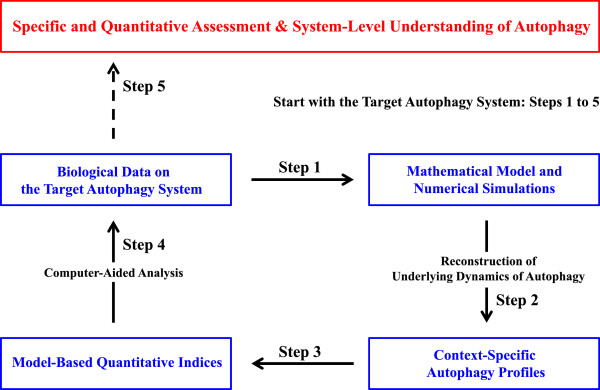
**Development of the experimental context-specific quantitative autophagy indices from the minimal model.** The workflow of the process is as follows: 1. Establishment of the minimal autophagy model for the target autophagy system. 2. Reconstruction of underlying dynamics of the autophagic process at good time resolution. 3. Introduction of two quantitative autophagy indices measuring autophagy activity based on the model-driven autophagy profile. 4. Computer-aided analysis of the biological data with the help of the indices. 5. Specific/quantitative interpretation and system-level understanding of the system.

The aim of the present study is to propose the experimental context-specific quantitative indices of the autophagy activity based on the minimal autophagy model and to apply the indices to the simulation-based analysis of the target autophagy system (see Figure 2). Specifically, based on the minimal autophagy model [31], we reconstruct underlying profiles of the autophagic process such as the time evolution of autophagy fluxes, levels of autophagosomes/autolysosomes, and corresponding cellular changes at good time resolution, from the biological experiments of the target autophagy system [12-14,26,32,33]. Then, two quantitative indices measuring autophagy activities in the induction of sequestration fluxes and in the selective degradation are proposed from the model-driven profiles. Finally, the biological experiments are interpreted with the help of the indices, not only to examine how the autophagy system responds to cellular damaging but also to prove the causal relationships among the steady-state autophagy levels, autophagic fluxes, and corresponding cellular changes in a specific and quantitative manner. Developing such quantitative autophagy indices from the minimal model will be highly challenging but indispensable for the assessment of autophagy activity and of its roles associated with different physiopathological states.

## Methods

### Model systems and simulations

We first outline briefly the minimal model used in developing quantitative indices of the autophagy activity [31]. In this model, the multi-step autophagic process is divided into autophagosome formation, autolysosome formation, and intralysosomal hydrolysis steps, and the intracellular ATP and amino acids are considered to be the key molecules serving as a bridge among the autophagic process, non-autophagic degradation, and protein/organelle synthesis (see Figure 1). The dynamics of the model system are described by eight coupled differential equations 1 to 6, which are solved via the 5th order Runge–Kutta method for very high precision. Since the present study is designed to analyze a specific autophagy mechanism rather than to examine the general principle of autophagy, we have focused on the target autophagy system in the parameter selection: The key parameters used in the simulations have been fixed in accord with carefully selected biological data of the target autophagy system [12-14,26,32-34]. A few adjustable parameters, which have been set to reflect the experimental context, have minor influence on the simulations (see Table 1).

**Table 1:** Parameters in the model

**Symbol**	**Definition**	** Unit**	**Value**	**Reference**
$r_{g}^{\left(0\right)}$	Rate constant for autophagosome formation (normal value)	s^− 1^	* 1.12 × 10^− 5^	[12], [13], [14]
*k*_*g*_	Constant for autophagosome formation (ATP dependency)	mM	* 4.01	[12], [14]
*p*_*g*_	Constant for autophagosome formation (ATP dependency)	mM	* 3.00	[12], [14]
*a*_*g*_	Constant for autophagosome formation (amino acids dependency)	mM	4.50	
*γ*_*g*_	Constant for autophagosome formation (amino acids dependency)	(unitless)	* 1.22	[13]
*ξ*_*g*_	Constant for autophagosome formation (amino acids dependency)	mM^− 1^	* 7.49 × 10^− 2^	[13]
*r*_*l*_	Rate constant for autolysosome formation	s^− 1^	* 2.47 × 10^− 5^	[12], [14]
*k*_*l*_	Constant for autolysosome formation (ATP dependency)	mM	* 4.01	[12], [14]
*p*_*l*_	Constant for autolysosome formation (ATP dependency)	mM	* 3.00	[12], [14]
*r*_*h*_	Rate constant for intralysosomal hydrolysis	s^− 1^	* 1.39 × 10^− 5^	[12], [14]
*δ*_*h*_	Exponent for intralysosomal hydrolysis (ATP dependency)	(unitless)	* 7.24 × 10^− 1^	[12], [14]
*k*_*h*_	Constant for intralysosomal hydrolysis (ATP dependency)	mM	* 2.99	[12], [14]
*r*_*s*_	Rate constant for protein/organelle synthesis	mM ⋅ s^− 1^	* 1.48 × 10^− 5^	[34]
*k*_*s*_	Constant for protein/organelle synthesis (amino acids dependency)	mM	* 1.77 × 10^1^	[34]
$C_{A}^{\left(m\right)}$	ATP concentration corresponding to maximal protein/organelle synthesis rate	mM	3.00	

Parameters with asterisks (*) are fixed, determined from biological experiments [12-14,34]. Those without asterisks are adjustable, depending on experimental setups.

### Dynamic equations

In this model, variations of the autophagosome concentration with time are determined by the difference between the autophagosome formation specific rate *R*_*gi*_ and the autolysosome formation specific rate *R*_*l*_. Denoting by *C*_*gi*_ the concentration of autophagosomes originating from proteins/organelles S_*i*_, we write the equations for the dynamics of autophagosomes in the form (*i* = 1, 2):

(1) $$\frac{dC_{\mathrm{gi}}}{\mathrm{dt}}=R_{\mathrm{gi}}C_{\mathrm{Si}}\;-\;R_{l}C_{\mathrm{gi}},$$

where *C*_*Si*_ represents the concentration of S_*i*_. Next, the intracellular concentration *C*_*li*_ of autolysosomes, originating from S_*i*_ is determined by the difference between the autolysosome formation specific rate *R*_*l*_ and the intralysosomal hydrolysis specific rate *R*_*h*_. The equations governing the dynamics thus read (*i* = 1, 2):

(2) $$\frac{dC_{\mathrm{li}}}{\mathrm{dt}}=R_{l}\left(t\;-\;\tau \right)\;C_{\mathrm{gi}}\left(t\;-\;\tau \right)\;-\;R_{h}C_{\mathrm{li}}$$

Note that the autolysosome concentration at time *t* is affected by the autophagosome concentration at time *t* − *τ*, earlier by the delay time *τ* which is taken to be 8 minutes (*τ* = 480 s) [26,32,33].

We have defined resident proteins/organelles S_1_ as the proteins and organelles which conduct normal functions in the cell, and assumed that they are translated from normal folding intermediates transcribed from DNA normally into RNA. On the other hand, by abnormal proteins/organelles S_2_, we have meant the proteins and organelles which conduct abnormal functions in the cell and assumed that they are made from two distinct sources: either from misfolded proteins and peptides, caused by genetic variants and mutations or intracellular conditions, or from resident proteins and organelles, damaged or aged by harmful conditions. Incorporating these, we have described the dynamics of S_1_ and of S_2_ by the evolution equations for the concentrations *C*_*S*1_ and *C*_*S*2_ of S_1_ and S_2_, respectively:

(3) $$\frac{dC_{S1}}{\mathrm{dt}}=\left(1\;-\;\alpha \right)R_{S}\;-\;R_{d1}\;-\;\beta C_{S1}\;-\;R_{g1}C_{S1}$$

(4) $$\frac{dC_{S2}}{\mathrm{dt}}=\alpha R_{S}\;-\;R_{d2}+\beta C_{S1}\;-\;{R_{g}}_{2}C_{S2},$$

where *R*_*S*_ represents the (total) protein/organelle synthesis rate (from DNA) and *α* is the fraction of S_2_ in the protein/organelle synthesis. Accordingly, S_1_ and S_2_ are produced at the rates of (1 − *α*)*R*_*S*_ and *αR*_*S*_, respectively. Further, *R*_*di*_ represents the non-autophagic degradation rates of S_*i*_ (for *i* = 1, 2) and *β* is the specific rate of deterioration of S_1_, i.e., transformation from S_1_ to S_2_.

The dynamics of intracellular amino acids, the concentration of which is denoted by *C*_*a*_, takes the form

(5) $$\frac{dC_{a}}{\mathrm{dt}}=\mu_{a}R_{h}\sum_{i=1}^{2}C_{\mathrm{li}}+\;\mu_{d}\sum_{i=1}^{2}R_{\mathrm{di}}+R_{a}\;-\;\mu_{s}R_{S},$$

of which the first and second term on the right-hand side correspond to the increase of amino acids due to intralysosomal hydrolysis and nonautophagic protein/organelle degradation, respectively, with appropriate constants *μ*_*a*_ and *μ*_*d*_ describing the mean numbers of amino acids produced from autophagic degradation and from non-autophagic degradation, respectively. The third term represents the net intracellular amino acid generation rate due to various intracellular metabolisms other than autophagy, and has been defined according to *R*_*a*_ = *μ*_*c*_*R*_*S*_ with an appropriate constant *μ*_*c*_. The last term stands for the reduction of amino acids due to protein/organelle synthesis, with the constant *μ*_*s*_ denoting the mean number of amino acids within a protein/organelle.

It has been assumed that the intracellular ATP concentration *C*_*A*_ increases due to intralysosomal hydrolysis and non-autophagic protein/organelle degradation via cytosolic and mitochondrial ATP production. The corresponding dynamic equation for ATP reads:

(6) $$\frac{dC_{A}}{\mathrm{dt}}=\nu_{a}R_{h}\sum_{i=1}^{2}C_{\mathrm{li}}+\nu_{d}\sum_{i=1}^{2}R_{\mathrm{di}}+R_{A}\;-\;\nu_{s}R_{S},$$

where *ν*_*a*_ and *ν*_*d*_ describe the mean numbers of ATP molecules produced from autophagic degradation and from non-autophagic degradation, respectively, and *ν*_*s*_ gives the mean number of ATP molecules consumed in unit protein/organelle synthesis. The net intracellular ATP generation rate *R*_*A*_ is given by the difference between the cytosolic and mitochondrial ATP production rate and the ATP consumption rate, and is assumed to be *R*_*A*_ = *ν*_*c*_*R*_*S*_ with a constant *ν*_*c*_.

For simplicity, we have supposed that an average protein/organelle in the virtual cellular system is composed of 500 amino acid residues, implying that 500 amino acids are consumed for the synthesis of a protein/organelle. Further, considering the fact that elongation of one amino acid during translation requires approximately four ATP molecules, we have assumed 2000 molecules of ATP involved in the synthesis of a protein/organelle. However, since the efficacy of protein/organelle recycling is expected to be less than 100%, the increases of amino acids and ATP due to intralysosomal hydrolysis or non-autophagic protein/organelle degradation should be less than 500 and 2000 molecules per degradation of one protein/organelle, respectively. In this study, we have chosen the base run parameter values as *μ*_*a*_ = *μ*_*d*_ = *ν*_*a*_ = *ν*_*d*_ = 300, *μ*_*c*_ = 200, *ν*_*c*_ = 1700, *μ*_*s*_ = 500, and *ν*_*s*_ = 2000.

## Results

### Quantitative indices from minimal models

**Figure 3:**
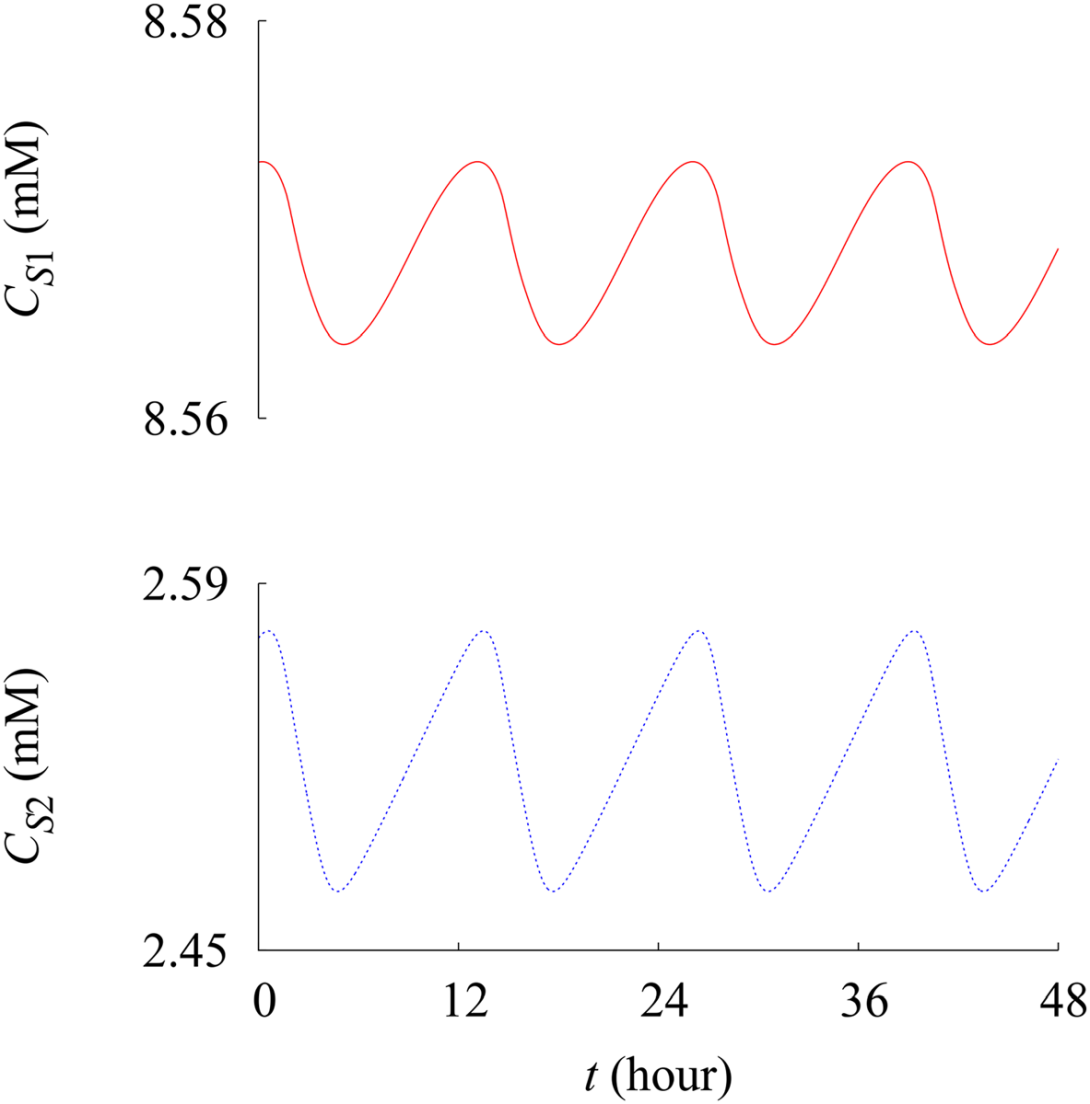
**Time evolution of the protein/organelle concentrations.** Red solid and blue dotted lines describe the time evolution of the concentrations *C*_*S*1_ (top) and *C*_*S*2_ (bottom) of resident and abnormal proteins/organelles, respectively. Data have been obtained at $r_{g}=r_{g}^{\left(0\right)}$ and *β* = *β*^(0)^.

Solving Eqs. 3 and 4, we have obtained the time evolution of concentrations *C*_*S*1_ and *C*_*S*2_ of resident S_1_ and abnormal proteins/organelles S_2_, respectively (see Figure 3). They display oscillations with the period of 12 h 55 min (or the natural frequency 0*.*0774 h^‒ 1^) [35-39], with ${\bar{C}}_{S1}$ and ${\bar{C}}_{S2}$ of the average values of *C*_*S*1_ and *C*_*S*2_ given by 8.57 mM and 2.52 mM, respectively.

Based on such model-driven profiles of intracellular concentrations of proteins/ organelles, we propose the fractional abnormal protein/organelle concentration *f*_*c*_ as a simple index of the cellular protein/organelle quality [40]:

(7) $$f_{c}\equiv \frac{{\bar{C}}_{S2}}{{\bar{C}}_{S1}+{\bar{C}}_{S2}},$$

which takes values between 0 and 1: While the value *f*_*c*_ ≈ 0 addresses that most of the proteins/organelles in the system are normal, *f*_*c*_ ≈ 1 indicates that the majority of the proteins/organelles are damaged.

**Figure 4:**
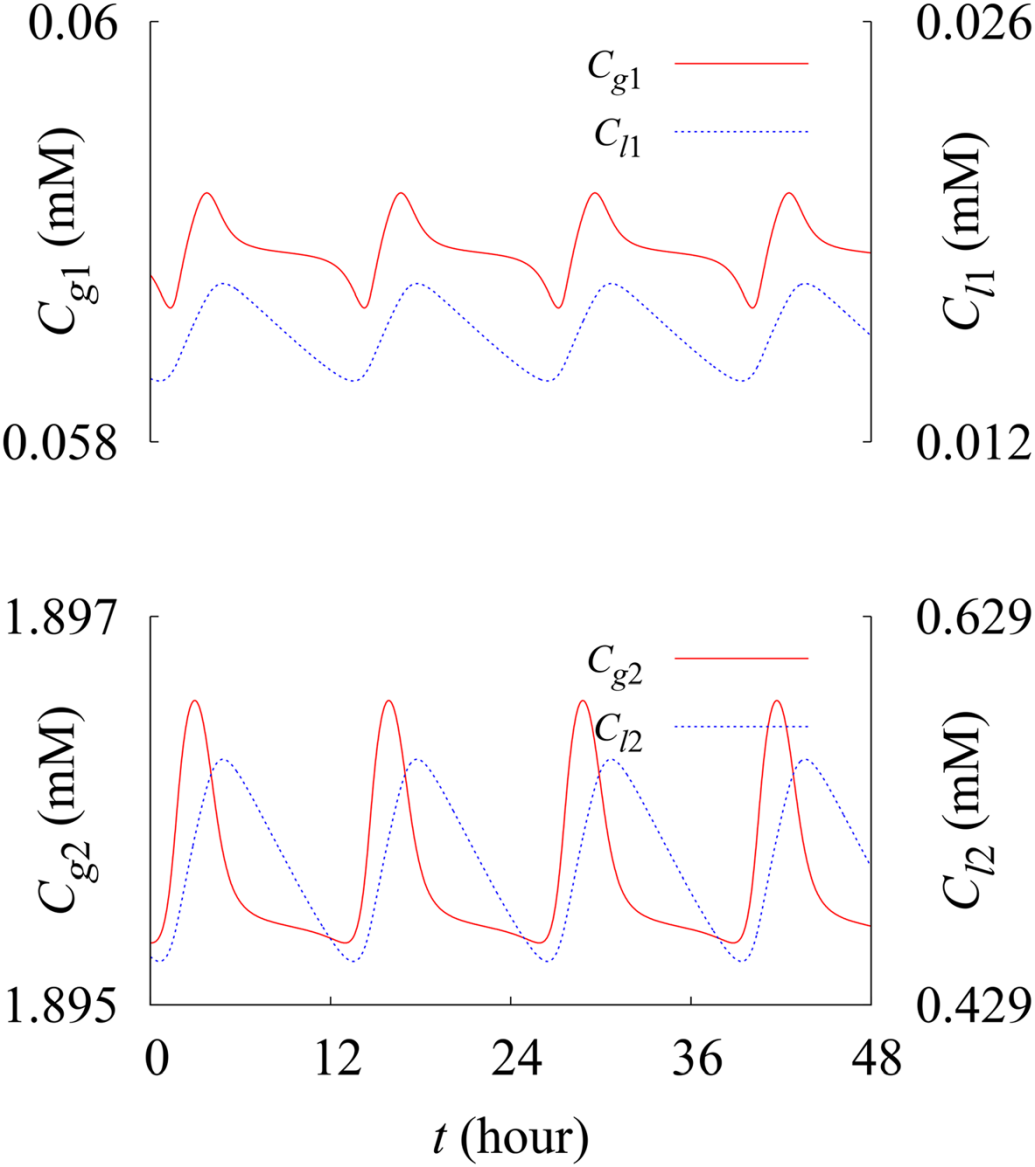
**Time evolution of the autophagosome and autolysosome concentrations.** Red solid and blue dotted lines describe the time evolution of the autophagosome concentrations *C*_*g*1_ and *C*_*g*2_ and autolysosome concentrations *C*_*l*1_ and *C*_*l*2_, respectively, transformed from S_1_ (top panel) and from S_2_ (bottom panel). Data have been obtained under the condition $r_{g}=r_{g}^{\left(0\right)}$ and *β* = 0.2 %/h ≡ *β*^(0)^*.*

Figure 4 exhibits the time evolution of concentrations *C*_*g*1_ and *C*_*g*2_ of autophagosomes from S_1_ and from S_2_, respectively (left axis), and the time evolution of concentrations *C*_*l*1_ and *C*_*l*2_ of autolysosomes from S_1_ and from S_2_, respectively (right axis).

It has turned out that they display oscillations with the period of 12 h 55 min (or the natural frequency 0*.*0774 h^‒ 1^) in the absence of external driving [41-48]. The percentile ratios of the peak-to-peak amplitudes to the mean levels of the oscillations are 0.929% for *C*_*g*1_, 0.0676% for *C*_*g*2_, and 20.8% for *C*_*l*1_ and for *C*_*l*2_, with negligibly small standard deviations (of the order 10^−5^ relative to the amplitudes). Note here that the oscillation amplitudes of autophagosome concentrations are far smaller than those of autolysosome concentrations. The average concentrations *C*_*g*1_ and *C*_*g*2_ turned out to be 0.0589 mM and 1.90 mM, respectively and those of autolysosomes, *C*_*l*1_ and *C*_*l*2_, to be 0.0156 mM and 0.503 mM, respectively.

Furthermore, the minimal autophagy model allows us to characterize quantitatively the relationship between the autophagy activity and the cellular change, based on the system-level understanding of the autophagic process at good time resolution, which may not be possible via the current in vivo and in vitro measurements. For example, from the model-driven profiles of the steady-state concentrations of autophagic intermediates (Figure 4), together with the dynamics of proteins/organelles (Figure 3), substrate selectivity of the autophagic process has been observed. There are significant differences between the production rates of autophagosomes/autolysosomes from resident proteins/organelles and those from abnormal proteins/organelles: The steady-state concentration ratio of *C*_*l*1_ to *C*_*S*1_ is given by 0.00182 whereas that of *C*_*l*2_ to *C*_*S*2_ is 0.200. In addition, the ratios of *C*_*g*1_ to *C*_*S*1_ and of *C*_*g*2_ to *C*_*S*2_ read 0.00687 and 0.754, respectively. For a more specific and quantitative analysis of various facets of the substrate selectivity of autophagy [40,49-54], we here propose a quantitative index of the autophagy selectivity (see Figure 1).

The autophagy selectivity index Φ_S_, which is designed to quantify the selective autophagic degradation of abnormal or resident protein/organelle, is given by:

(8) $$\Phi_{S}\equiv \frac{{\bar{C}}_{l2}}{{\bar{C}}_{S2}}-\frac{{\bar{C}}_{l1}}{{\bar{C}}_{S1}},$$

where ${\bar{C}}_{\mathrm{li}}$ denotes the average concentration of autolysosomes from S_*i*_(*i* = 1, 2). Note that positive values of Φ_S_ imply that autophagic degradation of S_2_ exceeds that of S_1_ whereas negative values correspond to the degradation of S_1_ exceeding that of S_2_.

**Figure 5:**
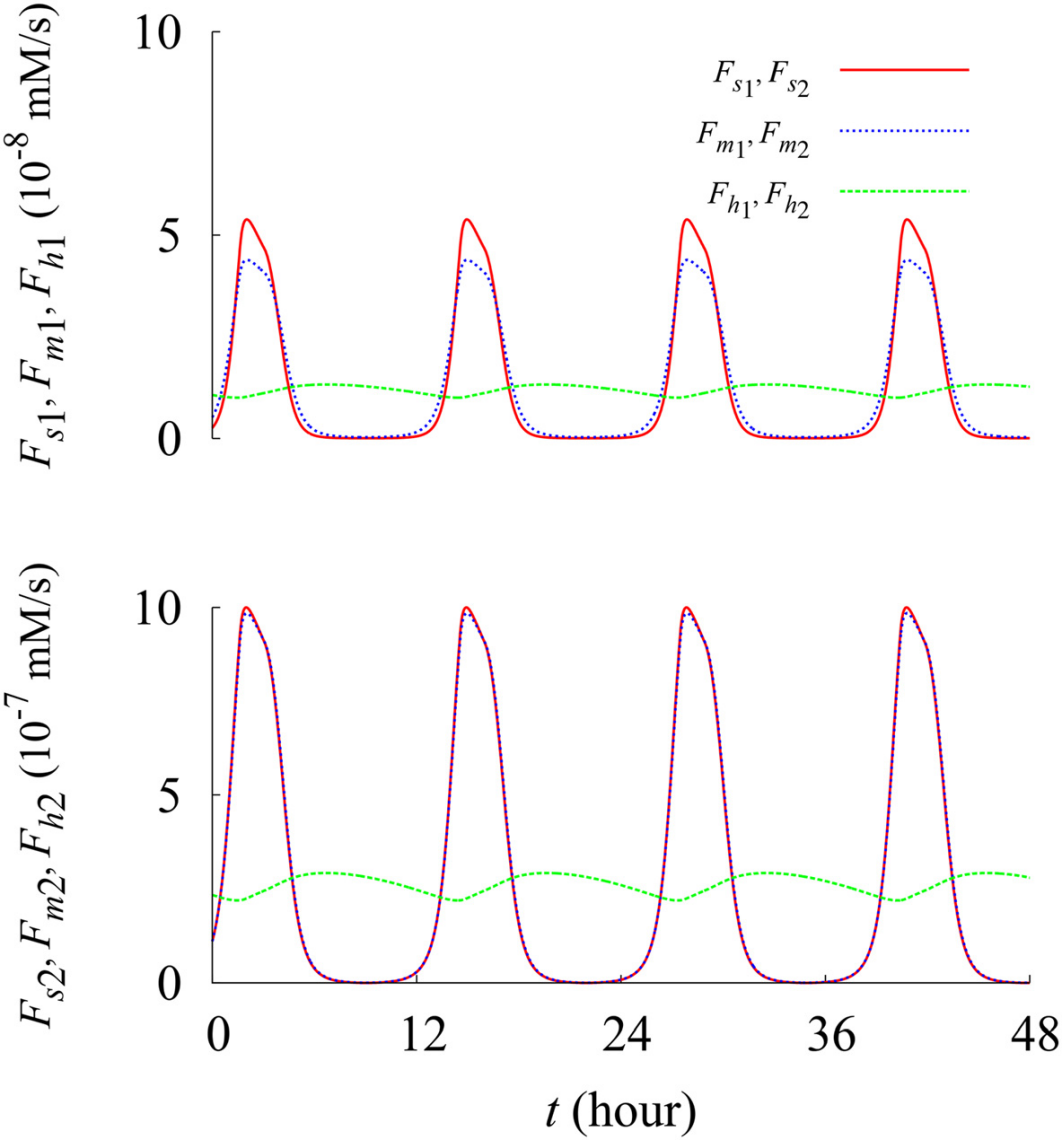
**Autophagic fluxes in sequestration, maturation, and intralysosomal hydrolysis.** Red solid, blue dotted, green dashed lines describe the time evolution of sequestration fluxes *F*_*s*1_ and *F*_*s*2_, maturation fluxes *F*_*m*1_ and *F*_*m*2_, and intralysosomal hydrolysis fluxes *F*_*h*1_ and *F*_*h*2_, respectively, originating from S_1_ (top) and from S_2_ (bottom). Data have been obtained at $r_{g}=r_{g}^{\left(0\right)}$ and *β* = *β*^(0)^.

This model allows us to reconstruct not only the dynamics of the steady-state concentrations of autophagosomes/autolysosomes but also the time evolution of autophagy fluxes. As shown in Figure 5, fluxes of sequestration, of maturation, and of intralysosomal hydrolysis from S_2_, denoted by *F*_*s*2_, *F*_*m*2_, and *F*_*h*2_, respectively, and those from S_1_, denoted by *F*_*s*1_, *F*_*m*1_, and *F*_*h*1_, display synchronized oscillations. Note also that the values of fluxes from S_2_ are much greater than those from S_1_.

Based on such comprehensive information as to the kinetics of the process obtained from the minimal model, we propose the autophagy induction index Φ_I_, which is devised to measure the induction of the total sequestration flux coming from both resident proteins/organelles S_1_ and abnormal proteins/organelles S_2_:

(9) $$\Phi_{I}\equiv \;{\bar{F}}_{s1}+{\bar{F}}_{s2},$$

where ${\bar{F}}_{\mathrm{Si}}$ denotes the average sequestration flux for S_*i*_(*i* = 1, 2).

### Quantitative analysis through the use of autophagy indices

To determine whether the indices given in the previous section are useful for the specific and quantitative assessment of the autophagy pathway and for the system-level understanding of the process, we in this section apply the indices to the interpretation of the target autophagy system, metabolically controlled autophagic protein/organelle degradation in the rat hepatocyte [12-14,26,32,33]. To be specific, we conduct the simulation-based analysis of how the autophagic process responds to cellular damaging and prove the causal relationships among the steady-state autophagy levels, autophagic fluxes, and corresponding cellular changes with the help of the indices.

**Figure 6:**
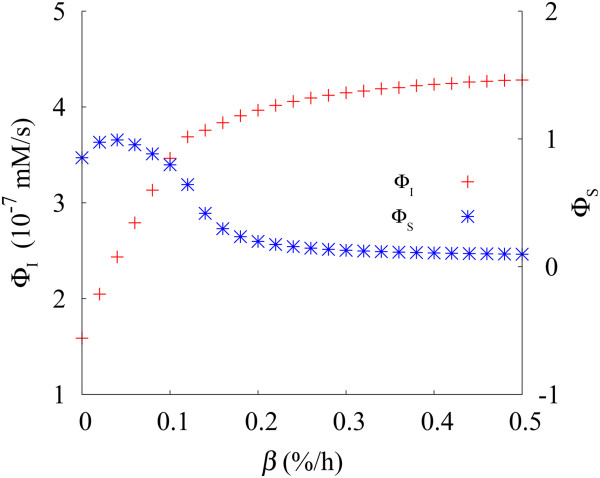
**Autophagy induction index Φ**_**I **_**(red pluses) and selectivity index Φ**_**S **_**(blue asterisks) versus the specific deterioration rate *β.*** Data have been obtained at $r_{g}=r_{g}^{\left(0\right)}$ with *β* varied up to 0.5 (%/h).

It is shown in Figure 6 that Φ_I_ grows drastically with the (specific) deterioration rate *β* until the value *β* ≈ 0.12, which illustrates autophagy induced against the cellular damaging rate. As *β* is raised beyond the value *β* ≈ 0.12, Φ_I_ increases gradually and displays a plateau. On the other hand, as *β* is increased from zero, Φ_S_ stays at relatively high positive values until *β* ≈ 0.12, beyond which it reduces gradually.

Therefore, both the non-selective mode of sequestration fluxes (represented by Φ_I_) and the selective mode of the autophagic degradation of abnormal proteins/organelles (described by Φ_S_) have been evaluated in a specific and quantitative manner with the help of the indices: The increasing behavior of Φ_I_ with the deterioration rate *β* suggests that the total sequestration flux coming from both resident and abnormal proteins/organelles is induced, resisting against the cellular damaging level. In addition, the positive values of Φ_S_ in the range of *β* from 0 to 0.5 (%/h) indicates that abnormal proteins/organelles are selectively removed via autophagy.

**Figure 7:**
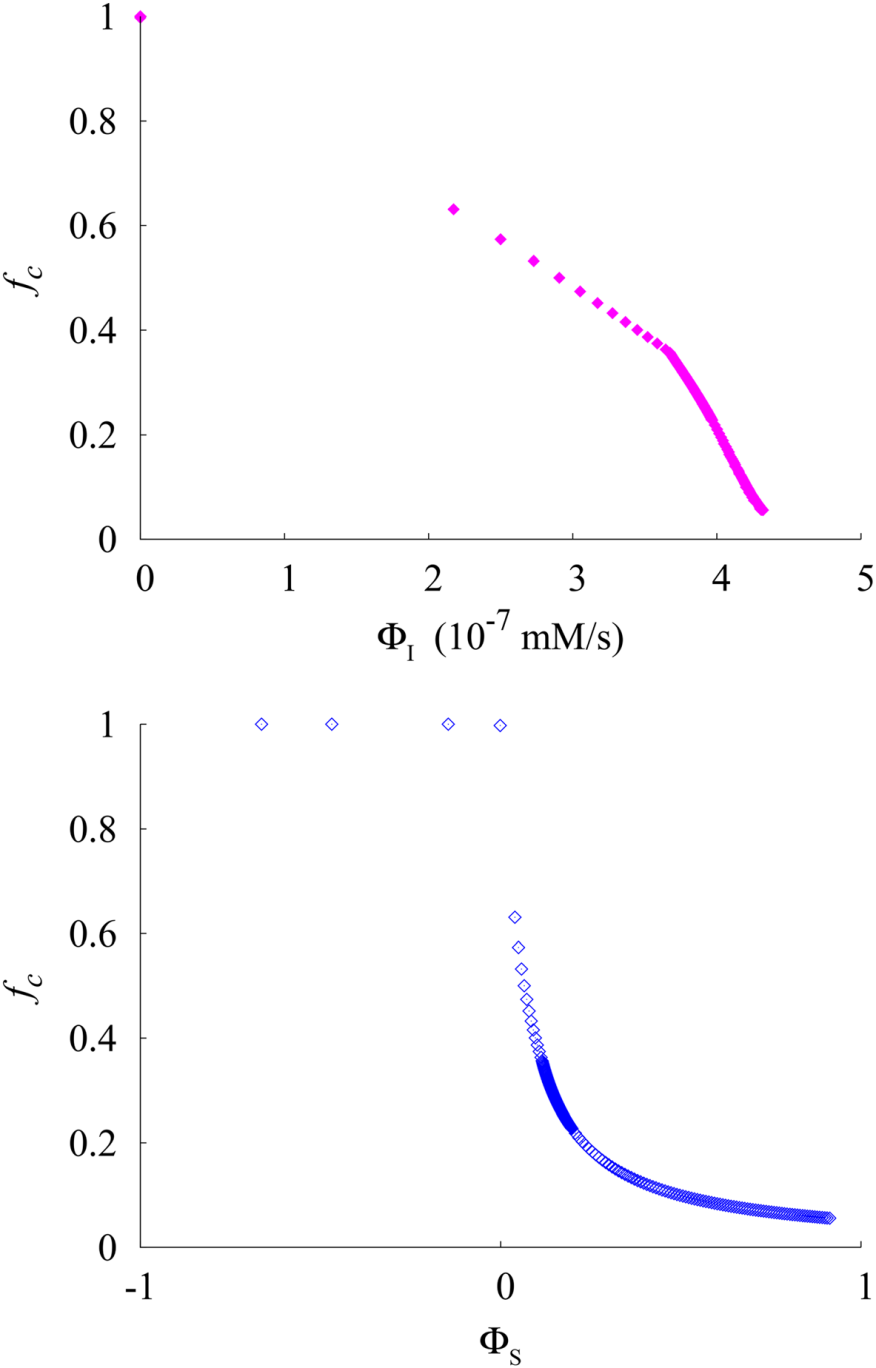
**Fractional protein/organelle concentration *f***_***c ***_**versus autophagy indices Φ**_**I **_**and Φ**_**S**_**.** Data plotted with filled purple rhombi (top panel) and open blue rhombi (bottom panel) have been obtained via suppressing or promoting autophagosome formation at *β* = *β*^(0)^. The rate constant has been raised from *r*_*g*_ = 0 to *r*_*g*_ = 10 at the increment of 0.01 (in units of $r_{g}^{\left(0\right)}$).

We then analyze how the promotion or suppression of autophagy affects the cellular quality control. Figure 7 exhibits the fractional abnormal protein/organelle concentration *f*_*c*_ depending on the autophagy indices Φ_I_ and Φ_S_, in response to varying the rate constant *r*_*g*_ for autophagosome formation in Eqs. A1 and A2 in Appendix A. Data have been obtained at the specific deterioration rate *β* = 0.2(%/h) ≡ *β*^(0)^, where the resident protein/organelle synthesis rate is approximately equal to the abnormal one.

In the case of no autophagic flux in the system (Φ_I_ = 0), the fractional concentration is positioned at a very high level ((*f*_*c*_ ≈ 1), indicating that most of the proteins/organelles in the system are damaged (top panel). As autophagosome formation is promoted, namely, as *r*_*g*_ is increased, however, Φ_I_ begins to increase. In particular, the abrupt increase in Φ_I_ at $r_{g}=r_{g}^{\left(1\right)}\approx \;0.03$, together with the drop in *f*_*c*_ at the same rate constant, appears here as the apparently discontinuous change in *f*_*c*_ at the values between Φ_I_ ≈ 0 and Φ_I_ ≈ 2. As Φ_I_ is increased further, *f*_*c*_ reduces to the normal level and drops eventually close to zero.

In addition, *f*_*c*_ varies also inversely proportional to Φ_S_. In case that the autophagic degradation of resident protein/organelle is larger than that of abnormal protein/organelle (Φ_S_ < 0), *f*_*c*_ stays at an abnormally high level (*f*_*c*_ ≈ 1), namely, most of the proteins/organelles in the system are damaged (bottom panel). As Φ_S_ is increased slightly above zero, there arises a discontinuous drop via which *f*_*c*_ becomes restored quickly to the normal level. As Φ_S_ is raised further and approaches unity, *f*_*c*_ keeps decreasing toward zero.

## Discussion

The minimal model for autophagy was originally developed to examine the dynamics of the autophagic process, describing specifically the rates at which autophagosome and autolysosome concentrations vary with time [31]. Based on the model, we have reconstructed underlying profiles of the autophagy process such as the time evolution of autophagy fluxes, levels of autophagosomes/autolysosomes, and corresponding cellular changes from the target autophagy system, in which the corresponding biological experiments [12-14,26,32,33] provide information only as to the changes before and after certain experimental perturbations on specific processes. We have then proposed quantitative indices of the autophagic process, and used them to analyze how the autophagic degradation compensates cellular damaging. Emphasis has been paid on the stressful conditions, specifically, at extremely high rates of protein/organelle deterioration. As discussed in Figures 6 and 7, indices Φ_I_ and Φ_S_ have successfully characterized the non-selective induction rate of autophagy and selective intralysosomal hydrolysis, respectively, in the presence of physiological perturbations such as variations of the cellular damaging rate and promotion or suppression of autophagosome formation. Furthermore, as shown in Figure 7, *f*_*c*_ reduces sensitively as Φ_I_ or Φ_S_ is increased; this might be inferred from the result that both the induction of the total sequestration flux, represented by Φ_I_, and the improvement of substrate selectivity in the autophagic degradation, described by Φ_S_, are beneficial for the control and regulation of the cellular protein/organelle quality, measured by *f*_*c*_. On the other hand, these results may disclose the roles of evolutionary-conserved basal autophagy in cell survival. Under extremely low levels of autophagic flux, the system may lose its ability to control the cellular protein/organelle quality, eventually resulting in cell death: As *r*_*g*_ is reduced below $r_{g}^{\left(1\right)}\approx 0.03$, the fractional concentration *f*_*c*_ remains at an abnormally high level, which may cause dysfunctions of the cell (Figure 7). Particularly, it is expected that once *r*_*g*_ reduces below $r_{g}^{\left(1\right)}$, normal cellular functions might not be recovered; this appears to be supported by the accompanying behaviors of resident and abnormal proteins/organelles as well as of autolysosomes, amino acids, and ATP (data not shown). Furthermore, in the case of no autophagic flux in the system as illustrated for *r*_*g*_ = 0, most of the proteins/organelles in the system are damaged (*f*_*c*_ ≈ 1), with the corresponding indices given by Φ_I_ = 0 and Φ_S_ < 0 (see Figure 7).

As the practical applications of this theoretical study, we remark implications of the quantitative autophagy indices for the development of a novel strategy for the assessment of autophagy. Several biochemical assays such as the measurement of autophagic sequestration [22], detection of the turnover of specific autophagic compartments or autophagy-related markers [23-25], and quantification of the autophagic protein/organelle degradation [55] have recently been suggested to provide indirect correlative data relating to the autophagic protein/organelle quality control. However, there still lacks full biochemical or molecular understanding of what distinguishes the selective and nonselective autophagic modes and how context- and activation-degree-dependency of selective/nonselective autophagy contributes to the protein/organelle quality control. Further, there are no absolute criteria, applicable to diverse situations, for determining the autophagic flux, mainly because some techniques and chemicals in certain assays are inappropriate, problematic, or may not work at all in other assays. Namely, it is not yet known whether the measurement of LC3-IIs/LC3-I and p62/SQSTM1 is generally applicable to other cell types, for changes in LC3-II or p62 amounts are tissue- and cell context-dependent; this constitutes the main caveat regarding the method. Also, the GFP-LC3 processing assay appears to depend on cell types and culture conditions, which is the main limitation. Even in the same assay, when using those techniques and chemicals, it is critical to consider the tissue- and cell context-dependent effects. Indeed, in some cases, the amounts of those indirect markers may not correlate well with the autophagosome/autolysosome accumulation detected by electron microscopy which is the most reliable criterion for autophagy activity.

In these circumstances, it is very desirable to have quantitative indices together with the appropriate mathematical model, which make it possible to provide a set of reliable criteria for the definition of the autophagy activation and further the assessment of its roles associated with different physiopathological states. Furthermore, the quantitative indices of the autophagy activation could give kinetic information as to the autophagic process, i.e., autophagosome formation, autolysosome formation, and intralysosomal hydrolysis. With such parameters available quantitatively, the worth of those conventional approaches to assessing autophagic fluxes or steady-state quantities of autophagosomes or autolysosomes could be greatly enhanced [27,56-61].

It is also to be noted that the proposed indices Φ_I_ and Φ_S_ are designed based on the selective profiles of autophagy, i.e., autophagic intermediates/fluxes from S_1_ and those from S_2_. Further, the indices can be modified depending on the experimental settings, with which selective autophagy is associated [40,49-54]: In fact, S_1_ and S_2_ in this study can denote different proteins/organelles in specific diseases such as ‘aging’ , ‘intracellular quality control and housekeeping’ , and ‘host defense against intracellular pathogens’. Accordingly, the indices should be easily applicable to various biochemical [20-25] and morphological [26-30] experiments, which selectively detect and quantify autophagy activity depending on their target substrates. Although such selective measurement of the autophagy activity with high specificity has not yet been carried out within current bioassay techniques [27,56,57], the indices should be useful in getting a better grasp of the substrate-selective autophagy activity and its role, complementing current biological techniques [20-30,55-57].

## Conclusion

We have proposed quantitative autophagy indices, based on the mathematical model, to define autophagy activity and further assess its role. With the help of the indices in conjunction with the computer-aided analysis, we have characterized quantitatively the cause-and-effect relationship among the steady-state autophagy levels, autophagic fluxes, and corresponding cellular changes in response to various physiological perturbations, which may not be probed via biological assays.

Our study acts as a natural link between experimental and computational/theoretical biology. Such an integrative approach should possibly lead to a comprehensive understanding of the control and/or regulatory mechanism of autophagy and reduce ambiguity as to causes and effects in the complex autophagy system. From a medical viewpoint, it should provide us new insight into the role of autophagy in various human diseases, including cancer, metabolic disorders, and neurodegenerative diseases and further help to develop new drugs or treatment methods which target specific autophagy pathways [16-19]. We hope to develop more realistic model-based quantitative indices of autophagy activity and new methods of monitoring autophagy, complementing recent biochemical assays; this is left for further study.

## Appendix A

### Autophagosome formation

Under normal conditions, it appears that abnormal proteins/organelles degrade preferentially via basal autophagy [49-52]. As the intracellular energy/nutrient reduces due to, e.g., starvation or increased metabolic demand, however, both resident and abnormal proteins/organelles are degraded non-selectively by bulk autophagy for the rapid supply of the essential energy/metabolite molecules. Therefore, it is assumed in this model that the autophagosome formation rate from resident proteins/organelles, which is lower than that from abnormal proteins/organelles under normal conditions, becomes gradually equal to that from abnormal proteins/organelles as the amino acid concentration is decreased [5,11,62,63]. Note, however, that the molecular mechanism of these steps is not included since the mechanism, via which ATP and amino acids control autophagosome formation, is relatively poorly understood. For example, preliminary studies show merely that amino acids regulate the LC3 level at the mRNA expression level [64]. Instead, we have obtained the dependence of these steps on intracellular ATP and amino acid concentrations, based on physiological-level experiments [12-14] (see Figure 1).

In consequence, we thus take the autophagosome formation specific rates *R*_*g*1_ (from resident proteins/organelles S_1_) and *R*_*g*2_ (from abnormal proteins/organelles S_2_) as functions of the intracellular concentrations *C*_*A*_ of ATP [12,14] and *C*_*a*_ of amino acids [13] in the form:

(A1) $$R_{g1}\left(C_{a},C_{A}\right)=r_{g}\frac{{C_{A}}^{4}}{{C_{A}}^{4}+{k_{g}}^{4}}\frac{{p_{g}}^{12}}{{C_{A}}^{12}+\;{p_{g}}^{12}}\frac{{a_{g}}^{8}}{{C_{a}}^{8}+\;{a_{g}}^{8}}\left(1+\gamma_{g}e^{-\xi_{g}C_{a}}\right),$$

(A2) $$R_{g2}\left(C_{a},C_{A}\right)=r_{g}\frac{{C_{A}}^{4}}{{C_{A}}^{4}+\;{k_{g}}^{4}}\frac{{p_{g}}^{12}}{{C_{A}}^{12}+\;{p_{g}}^{12}}\left(1+\gamma_{g}e^{-\xi_{g}C_{a}}\right),$$

where *r*_*g*_ is the rate constant for autophagosome formation, with appropriate constants *k*_*g*_, *p*_*g*_ (for ATP), *a*_*g*_, *γ*_*g*_, and *ξ*_*g*_ (for amino acids). In our simulations, the basal level of autophagy is suppressed or promoted by adjusting the value of *r*_*g*_ (in units of the normal value $r_{g}^{\left(0\right)}$) (see Table 1).

### Autolysosome formation

We next consider the autolysosome formation step, which consists of multiple fusions with lysosomes, which provide an acidic environment and a digestive function to the interior of the autophagosome [65,66]. In view of the experimental demonstration that the dynamics of autophagosome maturation depends on whether the ATP supply is on or off, we incorporate the intracellular ATP dependence of the step, and take the specific rate *R*_*l*_ in the form [12,14]:

(A3) $$R_{l}\left(C_{A}\right)=r_{l}\frac{{C_{A}}^{4}}{{C_{A}}^{4}+{k_{l}}^{4}}\frac{{p_{l}}^{12}}{{C_{A}}^{12}+\;{p_{l}}^{12}},$$

where *r*_*l*_ denotes the rate constant for autolysosome formation, with appropriate constants *k*_*l*_ and *p*_*l*_ for ATP. Note here that the possible difference between the maturation rates of autophagosomes from proteins/organelles S_1_ and S_2_[51,53], which relatively lacks proven molecular mechanism, has not been considered in this model. Although extensive characterization of ATG genes has yielded critical insight into the mechanism of autophagy activation and its flux, so far whether and how the selective fusion of autophagosomes to lysosomes is controlled remain poorly elucidated [50,51,54,67-70].

### Intralysosomal hydrolysis

The ATP dependency of the intralysosomal hydrolysis step, which displays relatively low sensitivity to the ATP concentration change compared with the autophagosome formation step, is incorporated. In accord with experiment [12,14], the intralysosomal hydrolysis specific rate *R*_*h*_ is taken as a function of the intracellular ATP concentration:

(A4) $$R_{h}\left(C_{A}\right)=r_{h}\frac{{C_{A}}^{\delta_{h}}}{{C_{A}}^{\delta_{h}}+{k_{h}}^{\delta_{h}}}$$

with appropriate exponent *δ*_*h*_ and constant *k*_*h*_ for ATP, where *r*_*h*_ is the rate constant for intralysosomal hydrolysis. Considering that there is little experimental evidence about the selective intralysosomal hydrolysis, we assume that the intralysosomal hydrolysis rates for autolysosomes originated from both S_1_ and S_2_ are the same.

### Protein synthesis and non-lysosomal degradation

In addition to the autophagic process, we incorporate the (total) protein synthesis rate *R*_*S*_, depending on the amino acid concentration *C*_*a*_, in agreement with experiment [34]. Assuming that the rate grows exponentially with the intracellular ATP concentration *C*_*A*_ increased to the steady-state value, we write the protein synthesis rate in the form:

(A5) $$R_{S}\left(C_{a},C_{A}\right)=\left\{\begin{array}{c} r_{s}\frac{C_{a}}{C_{a}+k_{s}}\frac{\exp\left[C_{A}\right]-1}{\exp\left[C_{A}^{\left(m\right)}\right]-1}\;\mathrm{for}\;C_{A}<C_{A}^{\left(m\right)}\\ r_{s}\frac{C_{a}}{C_{a}+k_{s}}\;\mathrm{for}\;C_{A}\;\geq \;C_{A}^{\left(m\right)} \end{array}\right)$$

with appropriate constant *k*_*s*_ for amino acid, where $C_{A}^{\left(m\right)}$ is the ATP concentration corresponding to the maximal protein/organelle synthesis rate and *r*_*s*_ denotes the rate constant for the protein/organelle synthesis.

Further, non-autophagic degradation machinery such as the ubiquitin-proteasome system has been considered in the model. We suppose that the amount of protein degradation by autophagy constitutes up to 80% of the total amount of protein degradation [71]. Taking the rate of non-autophagic degradation to be 25% of autophagic degradation, we have the rate of non–autophagic degradation (*i* = 1, 2):

(A6) $$\;R_{\mathrm{di}}=\frac{1}{4}R_{h}C_{\mathrm{li}}.$$

where *C*_*li*_ denotes the concentration of autolysosomes from S_*i*_.

## Abbreviations

ATP: Adenosine triphosphate; DNA: Deoxyribonucleic acid; RNA: Ribonucleic acid; mRNA: Messenger RNA; Atg: Autophagy-related gene; LC3: Microtubule-associated protein 1A/1B light chain 3A; LC3-II: LC3-phosphatidylethanolamine conjugate; LC3-IIs: Soluble form of LC3-II; GFP: Green fluorescent protein; GFP-LC3: GFP tagged LC3; p62: Nuclear pore complex (nucleoporins) p62; SQSTM1: Sequestosome 1.

## Competing interests

The authors declare that they have no competing interests.

## Authors’ contributions

KH designed the mathematical model, performed the simulations, analyzed the data, and wrote the manuscript. JK conceived of the study, participated in its design and coordination, and analyzed the data. MYC supervised the research, wrote the manuscript, designed the mathematical model, and analyzed the data. All authors read and approved the final manuscript.
